# Folate regulates RNA m^5^C modification and translation in neural stem cells

**DOI:** 10.1186/s12915-022-01467-0

**Published:** 2022-11-23

**Authors:** Xiguang Xu, Zachary Johnson, Amanda Wang, Rachel L. Padget, James W. Smyth, Hehuang Xie

**Affiliations:** 1 Epigenomics and Computational Biology Lab, Fralin Life Sciences Institute, Virginia Tech, Blacksburg, VA 24061 USA; 2 Department of Biological Sciences, College of Science, Virginia Tech, Blacksburg, VA 24061 USA; 3 Department of Biomedical Sciences and Pathobiology, Virginia-Maryland College of Veterinary Medicine, Virginia Tech, Blacksburg, VA 24061 USA; 4 Genetics, Bioinformatics and Computational Biology Program, Virginia Tech, Blacksburg, VA 24061 USA; 5 Graduate Program in Translational Biology, Medicine, and Health, Virginia Tech, Blacksburg, VA 24061 USA; 6 Fralin Biomedical Research Institute at VTC, Virginia Tech, Roanoke, VA 24016 USA; 7 Virginia Tech Carilion School of Medicine, Roanoke, VA 24016 USA

**Keywords:** RNA cytosine-5 methylation, Mouse neural stem cell, Folic acid, Polysome profiling, RNA bisulfite sequencing

## Abstract

**Background:**

Folate is an essential B-group vitamin and a key methyl donor with important biological functions including DNA methylation regulation. Normal neurodevelopment and physiology are sensitive to the cellular folate levels. Either deficiency or excess of folate may lead to neurological disorders. Recently, folate has been linked to tRNA cytosine-5 methylation (m^5^C) and translation in mammalian mitochondria. However, the influence of folate intake on neuronal mRNA m^5^C modification and translation remains largely unknown. Here, we provide transcriptome-wide landscapes of m^5^C modification in poly(A)-enriched RNAs together with mRNA transcription and translation profiles for mouse neural stem cells (NSCs) cultured in three different concentrations of folate.

**Results:**

NSCs cultured in three different concentrations of folate showed distinct mRNA methylation profiles. Despite uncovering only a few differentially expressed genes, hundreds of differentially translated genes were identified in NSCs with folate deficiency or supplementation. The differentially translated genes induced by low folate are associated with cytoplasmic translation and mitochondrial function, while the differentially translated genes induced by high folate are associated with increased neural stem cell proliferation. Interestingly, compared to total mRNAs, polysome mRNAs contained high levels of m^5^C. Furthermore, an integrative analysis indicated a transcript-specific relationship between RNA m^5^C methylation and mRNA translation efficiency.

**Conclusions:**

Altogether, our study reports a transcriptome-wide influence of folate on mRNA m^5^C methylation and translation in NSCs and reveals a potential link between mRNA m^5^C methylation and mRNA translation.

**Supplementary Information:**

The online version contains supplementary material available at 10.1186/s12915-022-01467-0.

## Background

Folate is an essential B-group vitamin and its synthetic form (folic acid) is widely used in nutrient supplements and fortified food [1]. As a major methyl donor, folate participates in the methylation of important biomolecules, such as DNA, RNA, proteins, and lipids, and in purine synthesis [2, 3]. Periconceptional maternal folic acid supplementation is a well-known preventative measure for neural tube defects (NTDs) in offspring [4–6]. Folate deficiency is associated with an increased risk of Alzheimer’s disease (AD) [7–9], while sufficient intake of folate reduces the risk of AD [10, 11]. Sufficient folate intake has also been observed to benefit cardiovascular health, including reducing the risk of stroke among adults with hypertension [12, 13]. Additionally, folic acid-supplemented diets have been demonstrated to prevent the transgenerational amplification of body weight in an obesity mouse model [14]. Despite the numerous benefits of folate supplementation, recent studies raised concern about the adverse effects of excess maternal folic acid supplementation. A recent study revealed a “U-shaped” correlation between maternal multivitamin supplementation frequency and an offspring’s risk of autism spectrum disorder (ASD) [15]. The adverse effects of excess maternal folate supplementation were further supported by a positive correlation found between the prescription of prenatal vitamins containing 1mg folic acid and the incidence of research-identified autism [16]. The U-shaped pattern of the influence of folate was corroborated in mouse model studies. Either a deficient or an excess folic acid supply during pregnancy causes comparable neurodevelopmental changes by delaying cerebral cortical neurogenesis [17]. High folate intake may lead to a higher incidence of ventricular septal defects, embryonic development delay, and short-term memory impairment in offspring [18–20]. Furthermore, negative effects of folate were demonstrated on the molecular level, as high maternal folate supplementation led to aberrant expression of autism-susceptible genes, including *Aust2* and *Fmr1* in the cerebral cortex of postnatal day 1 (P1) pups [21].

Prior to conception, maternal folate supplementation rescues the proliferation potential of neural stem cells (NSCs) in splotch (Sp^−/−^) embryos via epigenetic mechanisms [22]. The Sp^−/−^ mice have a homozygous mutation in the *Pax3* gene. Sp^−/−^ is a widely used neural tube defect (NTD)-prone mouse model with an impaired ability to synthesize thymidylate and undergoes a spontaneous occurrence of neural tube defects in its embryos [23, 24]. Recent studies showed that folic acid promotes the proliferation of neural stem cells [25–27] by increasing the phosphorylation of ERK1/2 [28] and activating ERK signaling, which is implicated in proliferation [29]. Notch signaling is triggered with the elevated expression of Notch1 and Hes5 on both the mRNA and protein levels [25]. Folate deficiency leads to an elevated level of homocysteine, which increases reactive oxygen species (ROS) production. This may induce DNA damage and result in apoptosis in NSCs [30]. In addition, homocysteine inhibits the phosphorylation of ERK1/2 and suppresses ERK signaling [31], which affects cell growth [29]. Protein expression levels and enzymatic activities of aconitase and respiratory complex III, two critical components in the mitochondrial respiratory chain, are decreased because of the neurotoxicity induced by homocysteine in NSCs [32]. Moreover, folic acid supplementation increases the protein expression and enzymatic activities of the DNMT family enzymes [26], resulting in an altered DNA methylation profile in the PI3K/AKT/CREB pathway [27]. High levels of homocysteine reduce protein expression and enzymatic activity of DNA methyltransferases, including DNMT1, DNMT3a, and DNMT3b [33]. This indicates that a dysregulation of methylation is an essential molecular mechanism underlying the pathogenesis of folate deficiency.

Post-transcriptional modification of RNA is emerging as a new layer of gene expression regulation [34]. Among numerous RNA modifications, 5-methylcytosine (m^5^C) is one of the most well-known in transfer RNAs (tRNAs), ribosomal RNAs (rRNAs), and, more recently, messenger RNAs (mRNAs) [35, 36]. m^5^C modification is essential for the regulation of diverse biological processes. m^5^C in tRNAs is involved in the regulation of tRNA stability and protein synthesis [37]. m^5^C in rRNAs affects the regulation of translational fidelity and ribosome biogenesis [38, 39]. m^5^C in mRNAs regulates the stability, nucleocytoplasmic export, and translational efficiency of mRNAs [40–43]. Folate affects the regulation of RNA m^5^C methylation as well. The one-carbon unit bound by folate has been shown to be essential for tRNA m^5^C methylation in mammalian mitochondria, which is required for mitochondrial mRNA (mt-mRNA) translation and subsequent oxidative phosphorylation [44]. Despite the critical roles of folate as a methyl donor, no previous study has investigated the influence of folate intake on mRNA m^5^C methylation.

This study aims to explore the link between folate dose-response and mRNA m^5^C methylation, transcription, and translation. We hypothesized that intake of the methyl donor folate influences RNA metabolism. To address this hypothesis, we systematically assessed the transcriptome-wide influence of folic acid deficiency and supplementation on RNA cytosine-5 methylation, transcription, and translation profiles in mouse neural stem cells.

## Results

### Effect of folate concentrations on mRNA abundance in mouse neural stem cells

**Fig. 1:**
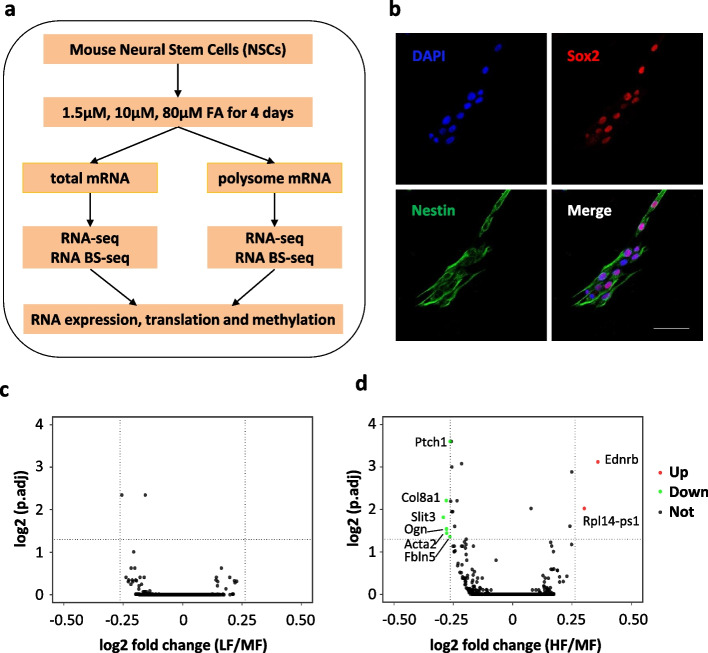
Influence of folate on mRNA abundance in NSCs. **a** Schematic representation of the experimental design. **b** Mouse NSC cultures were stained with the neural progenitor markers Nestin (cytoplasmic, green) and Sox2 (nuclear, red) and counterstained with DAPI (nuclei, blue). Scale bar: 50 μm. **c**, **d** Volcano plot showing the differentially expressed genes (adjusted *p*-value 0.05, fold change 1.2) in the comparisons of LF vs MF (**c**) and HF vs MF (**d**)

To generate gene expression profiles and transcriptome-wide maps of RNA m^5^C modification, we performed both RNA-seq and RNA bisulfite sequencing (RNA BS-seq) using total mRNA samples derived from mouse NSCs (Fig. 1a). Mouse NSCs were isolated from the subventricular zone (SVZ) and maintained as previously described [45]. To characterize NSCs in monolayer culture, we performed immunostaining using two NSC markers: Nestin and Sox2. Nestin is a cytoskeletal intermediate filament specifically expressed in neural stem cells [46] and Sox 2 is an HMG box transcription factor essential to maintaining the self-renewal of multipotent neural stem cells [47]. Double staining of Nestin and Sox2 showed that all cells were positive for both markers (Fig. 1b), indicating a highly homogenous neural stem cell culture. To investigate the dose-dependent influence of folate, NSCs were cultured in media with three different concentrations of folic acid for 4 days: 1.5 μM folic acid as the low folate concentration (LF), 10 μM folic acid as the median folate concentration (MF, folate level commonly supplied in cell culture media), and 80 μM folic acid as the high folate concentration (HF). With two biological replicates for each condition, six total mRNA-seq and six total mRNA BS-seq libraries were constructed and sequenced on the Hiseq 4000 platform in the 150-bp paired end mode.

An average of 26 million raw read pairs were generated for RNA-seq libraries with around 21 million read pairs uniquely mapped to the mouse reference transcriptome (Additional file 2: Table 1a). The gene expression levels calculated as transcripts per million (TPM) were provided (Additional file 3: Table 2). Pearson correlation analysis showed high reproducibility between the two biological replicates (Additional file 1: Fig. S1a-c). Principal component analysis showed that two replicates were clustered together (Additional file 1: Fig. S1d). Differential gene expression analysis was performed using DESeq2 [48]. Compared to MF controls, no differentially expressed gene (DEG) was identified in the LF condition (Fig. 1c) and only eight genes were found to be differentially expressed in the HF condition; however, these had only mild fold changes between 1.2 and 1.3 (Fig. 1d, Additional file 4: Table 3). Among the eight DEGs, 6 genes (Fbln5, Ogn, Ptch1, Acta2, Slit3, Col8a1) showed decreased mRNA levels and 2 genes (Ednrb, Rpl14-ps1) showed increased mRNA levels. The DEGs were validated by RT-qPCR (Additional file 1: Fig. S2a-g). These results indicate that folate deficiency or supplementation has subtle effects on mRNA abundance in NSCs.

### Total mRNA m^5^C profile in mouse neural stem cells

**Fig. 2:**
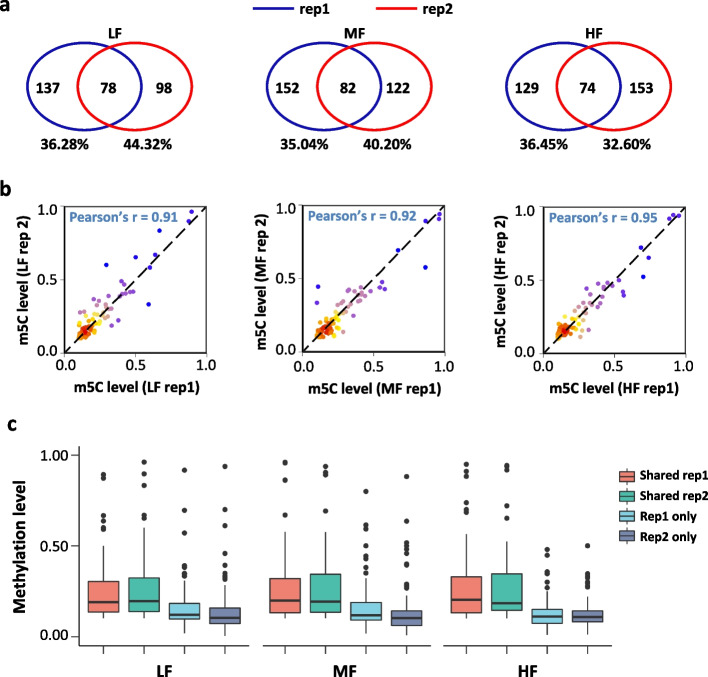
Characterization of high-confidence m^5^C sites in total mRNA BS-seq datasets. **a** Venn diagram showing the overlap of m^5^C sites between two replicates. **b** Dot plot showing the methylation level correlation of the overlapped m^5^C sites between two replicates. **c** Box plot showing the methylation level of overlapped and non-overlapped m^5^C sites in the two replicates

We generated an average of 230 million raw read pairs with around 160 million of those read pairs uniquely mapped to the reference genome for total mRNA BS-seq datasets (Additional file 2: Table 1b). Based on this spiked-in unmethylated mRNA control, the bisulfite conversion rates for the six RNA BS-seq libraries were determined to be above 99.9%. Sequential filtering steps were performed (see the “Methods” section) to remove potential false positive m^5^C sites. Good reproducibility was observed between the biological replicates. More specifically, 32.60 to 44.32% of m^5^C sites identified in one biological replicate were found to be methylated in the other biological replicate (Fig. 2a). In addition, the Pearson’s correlation of methylation level for m^5^C sites overlapped between the two replicates was in a range from 0.91 to 0.95 (Fig. 2b). The average methylation level of the overlapped m^5^C sites between biological replicates was higher than that of the m^5^C sites determined in one replicate alone (Fig. 2c). This suggests that the overlapped m^5^C sites between the two replicates were of high credibility, particularly with the stringent filtering steps applied in this study. Thus, the overlapped m^5^C sites between the two biological replicates were considered as high-confidence m^5^C sites for downstream analysis.

**Fig. 3:**
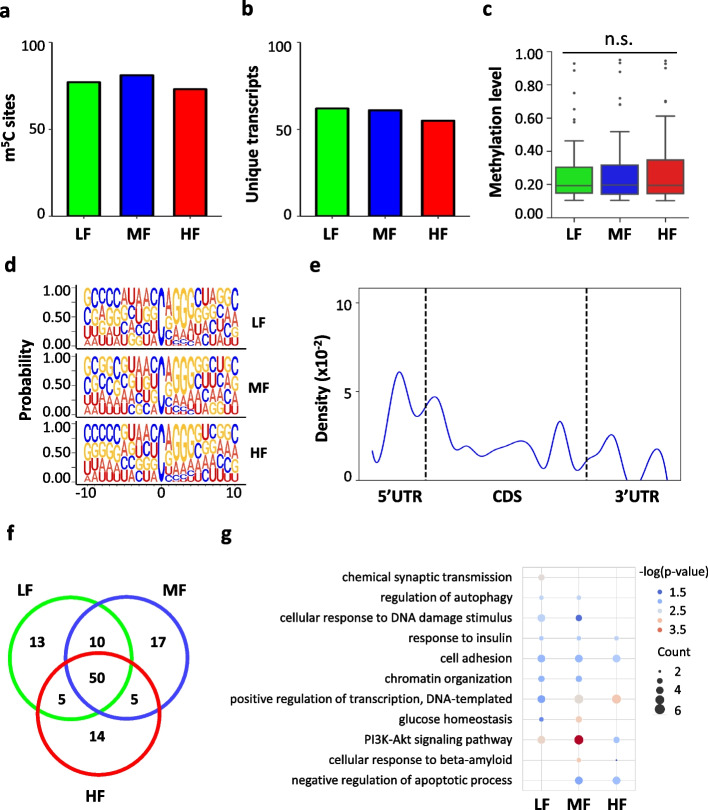
Distribution profile of m^5^C modification in total mRNAs of mouse NSCs. **a**, **b** Bar charts showing the numbers of m^5^C sites (**a**) and m^5^C-modified mRNAs (**b**) in total mRNA in NSCs. **c** Boxplot showing the methylation levels of m^5^C sites in total mRNAs in NSCs. **d** Sequence frequency logo for the sequence context proximal to m^5^C sites in total mRNA in NSCs. **e** Density plot showing the distribution of m^5^C sites along mRNA transcripts (5′UTR, CDS, 3′UTR). The moving average of percentages of mRNA m^5^C sites were shown. **f** Venn diagram showing the overlap of m^5^C sites among the three conditions (LF, MF, HF). **g** Bubble plot showing the GO terms of m^5^C-modified mRNAs in LF, MF, and HF

For NSCs cultured with different concentrations of folic acid, a total of 74 to 82 m^5^C sites within ~60 RNA molecules were identified (Fig. 3 a, b, Additional file 6: Table 5). The majority (96.7~98.3%) of these m^5^C sites were located on mRNAs and the remaining m^5^C sites were mapped to noncoding RNAs including pseudogenes (Additional file 1: Fig. S3a). Similar to previous reports [42, 49], the median methylation level of the m^5^C sites was approximately 20% among the three groups (19.3% in LF, 19.6% in MF, and 19.4% in HF) (Fig. 3c). The majority (71.8% in LF, 70.7% in MF, and 68.9% in HF) of the m^5^C sites were below 30%, and only 7.7–9.5% of the m^5^C sites showed methylation levels above 50% (Additional file 1: Fig. S3b). Additionally, analysis of the nucleotide composition matrix showed a dominant G-rich triplet (A/G-GGG) motif downstream of the m^5^C sites (Fig. 3d), which is similar to the sequence context reported in both human and mouse tissues and cell lines [42, 43, 49]. The distribution profile of the m^5^C sites in mRNAs showed an enrichment of m^5^C modification in 5′UTR, followed by an increasing peak of m^5^C sites immediately downstream of the translation initiation sites (Fig. 3e), indicating a potential role of m^5^C in the translational regulation of RNA metabolism [49, 50].

To investigate the influence of folate deficiency and supplementation on m^5^C profiles in NSCs, we first performed differential methylation analysis using Fisher’s exact test. After multiple-comparison adjustment, only one significant differentially methylated m^5^C site (DMS) was identified when comparing HF and MF, with mild methylation changes (Fbn1, chr2: 125321622, with a methylation level of 0.07 in HF, 0.11 in MF). We further plotted a Venn diagram to show the overlap of the m^5^C sites among the three conditions. It showed that the majority of the m^5^C sites were overlapped among the three conditions (64.10% in LF, 60.98% in MF, 67.57% in HF) (Fig. 3f). Gene Ontology (GO) annotation analysis of the m^5^C-modified mRNAs shared by the three conditions showed an enrichment for molecular functions and signaling pathways, including cell adhesion, response to insulin, and the PI3K-AKT signaling pathway (Fig. 3g). Cell adhesion and response to insulin are considered essential properties of neural stem cells [51, 52]. Notably, previous studies showed that folate is involved in the regulation of the PI3K-AKT signaling pathway in neural stem cells [27]. GO annotation analyses for the methylated transcripts in each condition revealed that m^5^C-modified mRNAs in LF showed enrichment for chemical synaptic transmission while m^5^C-modified mRNAs in MF and HF showed enrichment for negative regulation of apoptotic processes and cellular response to beta-amyloid. This shared and condition-specific functional enrichment supports the critical role of folate in neural stem cell metabolism.

### Folate induces changes in mRNA translation in a dose-dependent manner

**Fig. 4:**
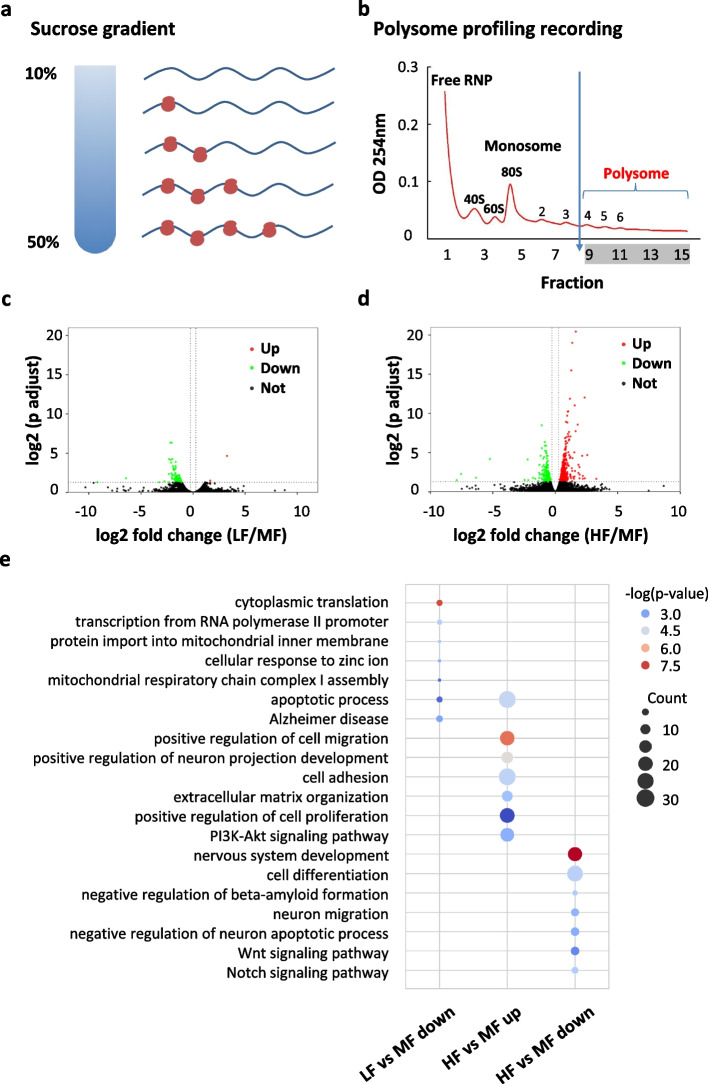
Folate deficiency and supplementation influence mRNA translation in NSCs. **a**, **b** Schematic representation of the sucrose gradient used to segregate ribosome-free and ribosome-bound RNAs (**a**) and representative polysome profile (1.5μM FA, replicate 1) recorded at 254 nm, polysome fraction (portion 9 to 15) is indicated (**b**). **c** Volcano plot showing the differentially translated genes (DTGs) in comparison between LF and MF. **d** Volcano plot showing the differentially translated genes (DTGs) in comparison between HF and MF. **e** Bubble plot showing the GO terms of the DTGs: decreased translation efficiency in LF (LF vs MF down), decreased translation efficiency in HF (HF vs MF down), and increased translation efficiency in HF (HF vs MF up)

To investigate the influence of folate on mRNA translation, we performed polysome profiling on the polysome-associated mRNAs. The cell lysates were separated by sucrose gradient ultracentrifugation and fractionated into 15 portions (Fig. 4 a, b). Polysome fractions with more than 3 ribosomes were pooled for RNA extraction, representing median to high actively translating mRNAs. The polysome RNA samples were digested with DNase enzyme to remove any residual DNA and then subjected to two rounds of oligo(dT) bead selection to enrich poly(A)-containing mRNAs. Polysome mRNA-seq libraries were prepared and sequenced on the Hiseq 4000 platform. An average of 28 million raw read pairs was obtained for the polysome mRNA-seq libraries with around 25 million read pairs uniquely mapped (Additional file 2: Table 1a). Pearson correlation analysis showed a high correlation between the two biological replicates (Additional file 1: Fig. S4a-c). Polysome profiling analysis was performed as previously described [53] to investigate the transcriptome-wide influence of folate on mRNA translation. We identified two genes with up-regulated translation efficiency and 94 genes with down-regulated translation efficiency in NSCs with folate deficiency (Fig. 4c, Additional file 5: Table 4), 148 genes with down-regulated translation efficiency, and 253 genes with up-regulated translation efficiency in NSCs with folate supplementation (Fig. 4d, Additional file 5: Table 4). Western blot was performed to validate the differentially translated genes (DTGs): Ndufa13 with decreased translation efficiency in LF showed reduced protein level in LF (Additional file 1: Fig. S5a), Gng11 with increased translation efficiency in LF showed increased protein level in LF (Additional file 1: Fig. S5b), and Ptx3 with increased translation efficiency in HF showed increased protein level in HF (Additional file 1: Fig. S5c). LF led to many more mRNAs with decreased translation efficiency, while HF led to more mRNAs with increased translation efficiency. This result indicates a dose-dependent influence of folate on mRNA translation.

GO annotation was further performed to determine the potential roles of these differentially translated genes (Fig. 4e). Down-regulated differentially translated genes in LF were enriched in cytoplasmic translation and cellular response to zinc ions. They also showed enrichment for mitochondrial function, such as protein localization to the mitochondrion and mitochondrial respiratory chain complex I assembly. A previous report indicated the role of folate metabolism in supporting mitochondrial DNA synthesis and oxidative phosphorylation [54]. Moreover, KEGG pathway analyses of these down-regulated differentially translated genes in LF showed enrichment in Alzheimer’s disease. Notably, previous studies showed that a low level of serum folate is associated with an increased risk of Alzheimer’s disease [7–9]. The down-regulated differentially translated genes in HF were enriched for nervous system development and critical neural functions, including cell differentiation, neuron migration, regulation of dendrite development, and negative regulation of the neuron apoptotic process. Down-regulated differentially translated genes in HF also showed enrichment for the Notch and Wnt signaling pathways, both of which are critical for NSC homeostasis [55, 56]. Up-regulated differentially translated genes in HF were enriched for the positive regulation of cell proliferation and the PI3K-AKT signaling pathway, which is in line with the finding that folate stimulates neural stem cell proliferation through the PI3K-AKT signaling pathway [27]. Recent reports showed that a mild and pervasive brain overgrowth induced by increased neural stem cell proliferation is one of the most common phenotypes in autism [57–59]. Such a difference in functional enrichment indicates the distinct impacts of folate deficiency and supplementation on the growth and differentiation of neural stem cells.

### Folate induces changes in polysome mRNA m^5^C methylation

**Fig. 5:**
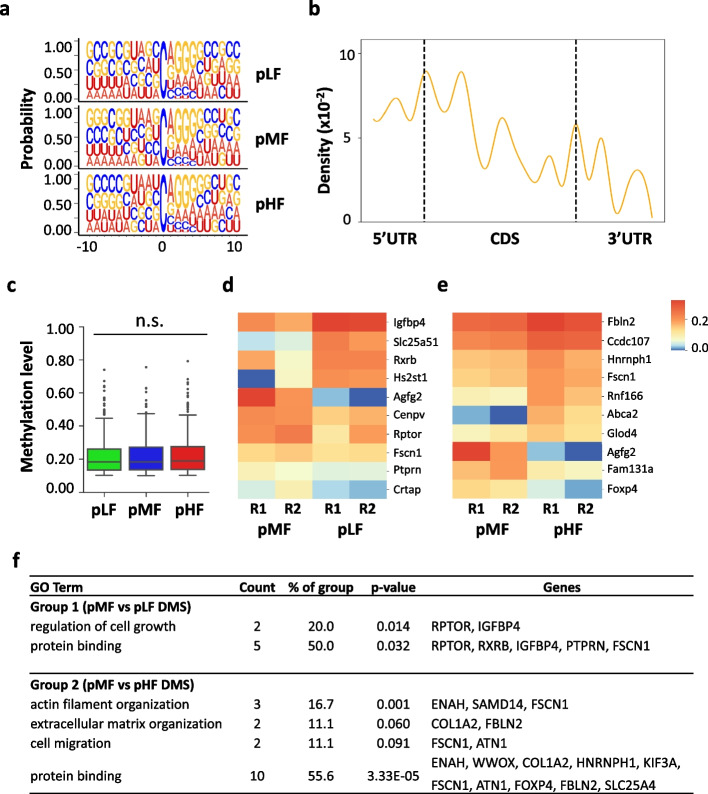
Influence of folate on polysome mRNA m^5^C methylation in mouse NSCs. **a** Sequence frequency logo for the sequence context proximal to m^5^C sites in polysome mRNAs. **b** Density plot showing the distribution of m^5^C sites along mRNA transcripts (5′UTR, CDS, 3′UTR). The moving average of the percentage is shown. **c** Boxplot showing the methylation levels of m^5^C sites in polysome mRNAs. **d** Heatmap showing the methylation level of differentially methylated m^5^C sites in comparison between pMF and pLF. Two biological replicates (R1, R2) are shown. **e** Heatmap showing the methylation level of differentially methylated m^5^C sites in comparison between pMF and pHF. Two biological replicates (R1, R2) are shown. **f** GO annotation of DMS-modified mRNAs of pMF vs pLF and pMF vs pHF. Representative GO terms are shown in the table

To provide direct evidence of the methylation status for actively translating mRNAs, we also prepared RNA BS-seq libraries using polysome mRNA samples and applied the same analytical pipeline used for the total mRNA BS-seq data on the polysome mRNA BS-seq datasets. We obtained an average of 285 million raw read pairs with around 137 million read pairs uniquely mapped to the reference genome (Additional file 2: Table 1b). Similar to the total mRNA BS-seq datasets, the spiked-in unmethylated Xef mRNA control showed a high bisulfite conversion rate (>99.9%) in the six polysome mRNA BS-seq libraries (Additional file 2: Table 1b). The biological replicates showed good reproducibility with methylation correlations ranging from 0.76 to 0.84 for the m^5^C sites overlapped between replicates (Additional file 1: Fig. S6a&b). The average methylation level of the overlapped m^5^C sites was higher than that of the m^5^C sites identified in one replicate only (Additional file 1: Fig. S6c). With the stringent filtering pipeline, we obtained 132 to 150 high-confidence m^5^C sites within 119 to 139 RNA molecules, where most of the m^5^C sites were located on mRNA molecules (pLF: 98.3%; pMF: 97.6%; and pHF: 97.8%) (Additional file 1: Fig. S6d, Additional file 6: Table 5). The median methylation level is around 19% (18.6% in pLF, 18.6% in pMF, and 19.0% in pHF) (Fig. 5c, Additional file 1: Fig. S6e). The sequence logo showed a similar m^5^C-A/G-GGG sequence context (Fig. 5a), and the distribution of m^5^C modification showed enrichment at 5′UTR and CDS, with an increasing peak at both the translation initiation and translation termination sites (Fig. 5b). Overall, the profile of polysome mRNA m^5^C sites resembled that of the total mRNA m^5^C sites in mouse neural stem cells.

We plotted a Venn diagram to check the overlap of the m^5^C sites among the three groups. More than half of the m^5^C sites were overlapped among the three groups (Additional file 1: Fig. S7a). Functional annotation of these m^5^C-modified mRNAs showed shared enriched GO terms among the three conditions, including cell adhesion, response to insulin, and the PI3K-Akt signaling pathway (Additional file 1: Fig. S7b). Alzheimer’s disease was also enriched in the three conditions (Additional file 1: Fig. S7b), with an increasing count of genes from high folate to low folate. This result suggests a potential role of folate deficiency in the progression of Alzheimer’s disease. m^5^C-modified mRNAs in LF and HF showed enrichment for in utero embryonic development (Additional file 1: Fig. S7b). which is supported by a previous report that both deficient and excess folate influence brain development [17].

To further determine the influence of folate on m^5^C modification in polysome-associated mRNAs, we performed Fisher’s exact test to identify differentially methylated m^5^C sites. We identified 10 DMS sites in the comparison between pLF and pMF, with 4 DMS sites being hypermethylated and 6 DMS sites being hypomethylated in pLF (Fig. 5d). In addition, 19 DMS sites were identified in the comparison between pHF and pMF, with 15 DMS sites being hypermethylated and 4 DMS sites being hypomethylated in pHF (Fig. 5e). GO annotation was performed to characterize the functions of the two sets of DMS-modified genes (Fig. 5f). Among the 10 DMS-modified mRNAs in the comparison between pLF and pMF, Rptor and Igfbp4 were involved in the regulation of cell growth. Among the 19 DMS-modified mRNAs in the comparison between pHF and pMF, Fscn1 and Atn1 played a role in the regulation of cell migration, and, Col1a2 and Fbln2 were involved in an extracellular matrix organization. In summary, the above results indicate that folate deficiency and supplementation influence polysome mRNA m^5^C methylation and that this influence is linked to critical neural functions.

### Correlation between m^5^C modification and mRNA translation

**Fig. 6:**
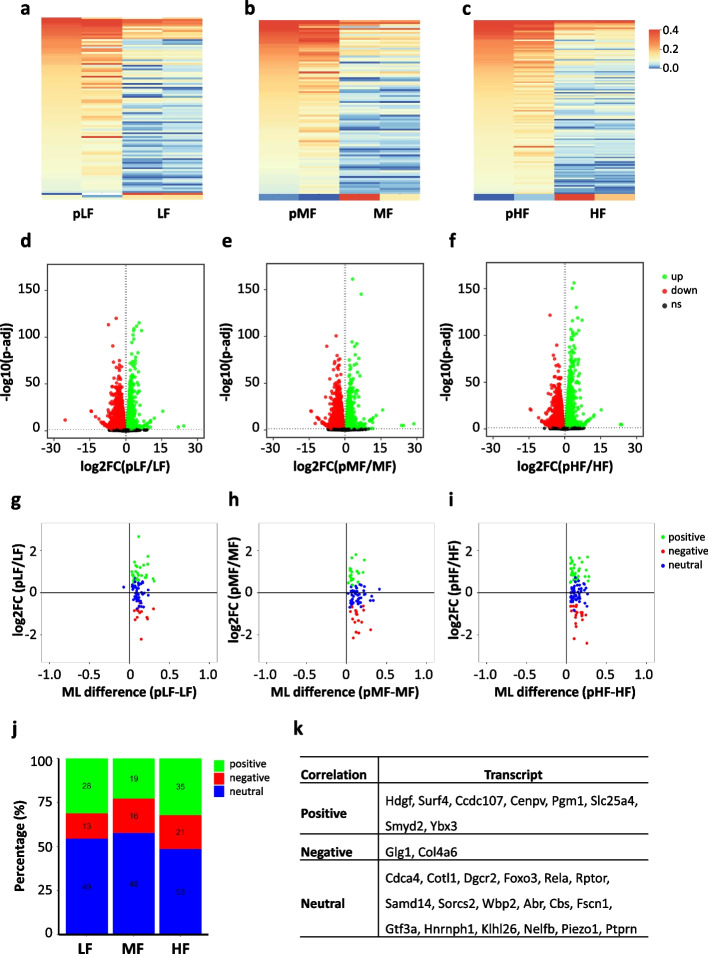
Correlation between m^5^C methylation and mRNA translation. **a**–**c** Heatmap showing the methylation profile of differentially methylated m^5^C sites in the comparison between total and polysome mRNAs. **d**–**f** Volcano plot showing the differentially expressed genes (DEGs) in the comparison between total and polysome mRNAs. **g**–**i** Distribution of mRNAs with a significant change in m^5^C methylation level in three categories: positive correlation, negative correlation, and neutral correlation between m^5^C methylation and mRNA translation efficiency. **j** Stacked bar charts showing the distribution of sites in different m^5^C methylation-mRNA translation correlation categories. **k** A summary table showing the transcripts with consistently positive/negative/neutral correlations between m^5^C methylation and mRNA translation among the three conditions.

Despite the similar profiles between total mRNA methylomes and polysome mRNA methylomes, more m^5^C sites were identified in polysome mRNAs. This result motivated us to perform a further analysis on the methylation differences between total and polysome mRNAs. Around half of the m^5^C sites in total mRNAs were overlapped with the m^5^C sites identified in polysome mRNAs (Additional file 1: Fig. S8a). To ensure comparable coverage between the two sets of methylomes, m^5^C sites included for further comparison had to meet two requirements: (1) high-confidence m^5^C sites in either the total or polysome mRNA methylomes and (2) a coverage depth ≥ 20 in both conditions. The comparison showed significant hypermethylation in polysome mRNAs (LF vs pLF, MF vs pMF, HF vs pHF) (Additional file 1: Fig. S8b). Hypermethylation in polysome mRNAs indicates m^5^C modification plays a role in the regulation of mRNA translation. In addition, a panel of differentially methylated m^5^C sites were identified in the comparison of LF vs pLF (99 DMS sites with 96 sites hypermethylated in pLF) (Fig. 6a), MF vs pMF (89 DMS sites with 88 sites hypermethylated in pMF) (Fig. 6b), and HF vs pHF (117 DMS sites with 116 sites hypermethylated in pHF) (Fig. 6c).

To further explore the relationship between m^5^C modification and mRNA translation, we performed correlation analyses by integrating the RNA expression data and the RNA methylome data generated from both total and polysome mRNAs. Differential expression analyses between total and polysome mRNA-seq datasets were performed. We divided the transcriptome into three categories based on the translation efficiency: up-regulated mRNAs in polysomes indicating high translation efficiency, no significantly changed mRNAs indicating median translation efficiency, and down-regulated mRNAs in polysomes indicating low translation efficiency. As such, we identified 7128 up-regulated transcripts and 7379 down-regulated transcripts in the comparison between pLF and LF (Fig. 6d), 6970 up-regulated transcripts and 7036 down-regulated transcripts in the comparison between pMF and MF (Fig. 6e), and 7285 up-regulated transcripts and 7262 down-regulated transcripts in the comparison between pHF and HF (Fig. 6f). Based on the translation status of mRNAs that carry DMS sites, the correlation between m^5^C modification and mRNA translation was grouped into three categories: positive (mRNAs with hypermethylated m^5^C sites and high translation efficiency, or mRNAs with hypomethylated m^5^C sites and low translation efficiency), negative (mRNAs with hypermethylated m^5^C sites and low translation efficiency, or mRNAs with hypomethylated m^5^C sites and high translation efficiency), or neutral (mRNAs with either hypermethylated or hypomethylated m^5^C sites and median translation efficiency) (Fig. 6 g–i). Around half of the mRNAs bearing DMS sites showed neutral correlations (54.44% in LF, 57.83% in MF, and 48.62% in HF), a quarter to one-third showed positive correlations (31.11% in LF, 22.89% in MF, 32.11% in HF) and the remaining one fifth showed negative correlations (14.44% in LF, 19.28% in MF, and 19.27% in HF) (Fig. 6 j). We further checked whether the diverse correlation relationship was transcript-specific. We found that 8 transcripts (Hdgf, Surf4, Ccdc107, Cenpv, Pgm1, Slc25a4, Smyd2, Ybx3) showed consistently positive correlations, 2 transcripts (Glg1, Col4a6) showed consistently negative correlations, and 18 transcripts (Cdca4, Cotl1, Dgcr2, Foxo3, Rela, Rptor, Samd14, Sorcs2, Wbp2, Abr, Cbs, Fscn1, Gtf3a, Hnrnph1, Klhl26, Nelfb, Piezo1, Ptprn) showed consistently neutral correlations among the three conditions (Fig. 6k). The correlation between m^5^C methylation and mRNA translation was visualized in IGV browser for Hdgf (positive correlation), Glg1 (negative correlation), and Dgcr2 (neural correlation) (Additional file 1: Fig. S9). The results revealed a transcript-specific relationship between m^5^C modification and mRNA translation.

## Discussion

The beneficial effect of folate supplementation before and during pregnancy has been known for almost three decades [60]. To prevent certain birth defects such as NTDs, folic acid fortification in the form of enriched grain food has been a regular practice in the USA since 1998 [61]. As a methyl donor, folate influences DNA methylation and gene expression [2, 21, 62]. To investigate the impact of folate on the epitranscriptome, this study provided transcriptome-wide profiles of mRNA abundance, translation, and m^5^C modification in both total mRNAs and polysome mRNAs from NSCs cultured in different concentrations of folate. Considering that NTDs may occur at a very early stage of brain development, NSCs were cultured for a relatively short period of time in this study. Thus, the influence of suboptimal folate concentration might not be fully represented yet during this period. Previous studies have shown that, compared to tissue samples, cells in culture tend to have fewer m^5^C sites determined [49, 63]. Using stringent criteria for methylation calling, this study focused on a small set of m^5^C sites with high confidence [64]. Therefore, some true m^5^C sites might be excluded during the filtering steps. Recently, Liu et al. have implemented an elegant tool to explore the isoform-level m5C sites across different tissues [65]. The focus of this study is to understand mRNA methylation, expression, and translation at the level of transcript. The short sequencing reads generated may not be ideal for the detection of all possible RNA isoforms. Despite these limitations, novel information was gathered with RNA-seq and RNA BS-seq using total poly(A) RNA samples isolated from NSCs cultured in low, median, and high folate media.

Although very few genes showed differential expression, hundreds of differentially translated genes were determined in the comparison between the control and folate deficiency or supplementation. Differentially translated genes between LF and MF were enriched in mitochondrial functions and linked to Alzheimer’s disease, while the differentially translated genes between HF and MF were enriched in the regulation of nervous system development and essential neural functions, including cell proliferation, differentiation, and apoptosis. Pathway analysis showed that the differentially translated genes between HF and MF are enriched in the Notch, Wnt, and PI3K-AKT signaling pathways, which play a critical role in the proliferation, differentiation, and survival of neural stem cells [55, 56, 66]. The enhanced PI3K-AKT signaling pathway is linked to the hyperproliferation of NSCs and brain overgrowth, which is considered as the common phenotype in autism [57, 59]. The differences in the functional enrichment indicate the distinct impacts of folate deficiency and supplementation on the growth and differentiation of neural stem cells.

RNA bisulfite sequencing of polysome-associated mRNAs has provided direct evidence of the methylation status of actively translating mRNAs. Interestingly, our study showed consistent hypermethylation in polysome mRNAs than that in total mRNAs, indicating a critical role of m^5^C modification in the regulation of mRNA translation. Integrative analysis showed that the correlation between mRNA m^5^C methylation and mRNA translation could be positive, negative, or neutral. Further examination indicated a transcript-specific relationship between m^5^C modification and mRNA translation. Differential methylation analyses showed that only one DMS site was identified in total mRNA methylomes, and a small set of DMS sites were identified in polysome mRNA methylomes. For both total mRNA methylomes and polysome mRNA methylomes, more than half of the high-confidence m^5^C sites were shared by the three conditions. Functional annotation of these m^5^C-modified mRNAs showed shared functions such as cell adhesion and response to insulin, and condition-specific enrichment, such as chemical synaptic transmission in LF. Interestingly, genes associated with Alzheimer’s disease are enriched in the three conditions in polysome mRNAs, with an increasing count of genes from high folate to low folate. Combined with previous reports in human studies [7–9] and the result that the down-regulated differentially translated genes in LF were enriched in Alzheimer’s disease, further studies are needed to investigate the effect of folate deficiency and supplementation in mouse models and in human populations.

## Conclusions

Our study identifies a transcriptome-wide influence of folate on mRNA m^5^C methylation and translation in NSCs and reveals a potential link between mRNA m^5^C methylation and mRNA translation. The mechanism underlying the regulation of mRNA translation by m^5^C methylation is worth of further research using in vivo models.

## Methods

### Mouse neural stem cell (NSC) culture and treatments

Mouse NSCs were isolated from the subventricular zone (SVZ) of the lateral ventricles as described previously [45]. NSCs were seeded on poly-ornithine and laminin-coated plates and the cells were incubated for four days with a media change at day 2. Cells were divided into three treatment groups based on the concentration of folic acid (FA): low FA group (LF, 1.5 μmol/L), medium FA group (MF, 10 μmol/L), and high FA group (HF, 80 μmol/L). LF media was prepared by mixing FA-free DMEM (Sigma, cat# D2429-100mL) and Ham’s F12 media (Fisher, cat# 11-765-054, containing 3 μmol/L FA) at a 1:1 volume ratio, supplemented with 2% B27, 2 mmol/L l-glutamine, 1× penicillin-streptomycin, 20 ng/mL epidermal growth factor (EGF, PeproTech, cat# AF-100-15), and 20 ng/mL basic fibroblast growth factor (bFGF, PeproTech, cat# 100-18B). 10mM FA stock was prepared from folic acid powder (Fisher, cat# AAJ6293706) and filtered through a 0.22-μm membrane.

### Polysome fractionation

Polysome fractionation was performed as previously described [67]. After treating with different concentrations of folic acid for 4 days, monolayer cultures of NSCs were incubated with cyclohexamide (CHX, 100 μg/mL, Sigma Aldrich, cat# 239765) at 37 °C for 10 min to stabilize ribosomes. After washing with ice-cold 1× phosphate-buffered saline (PBS) containing 100 μg/mL CHX, the NSCs were detached from the plate using a cell scraper and spun down at 300g at 4°C for 5min. The cell pellet was immediately transferred to a −80 °C freezer and stored for later analysis. Frozen cell pellets were thawed on ice, lysed in hypotonic lysis buffer, and centrifuged at 15,000 rpm for 5 min at 4 °C. The supernatant was collected and subjected to the OD260nm measurement using a Nanodrop 2000. Based on the values of OD260nm, an equal amount of lysate was loaded onto 10–50% sucrose gradients and ultra-centrifuged at 35,000 rpm at 4°C for 3 h using a SW41Ti rotor (Beckman Coulter). The gradients were fractionated into 15 fractions using Gradient Station (BioCamp). Polysome fractions (fractions 9–15) were identified, pooled, and extracted with TRIzol LS reagent. The purified polysome RNA samples were subjected to two rounds of oligo(dT) bead selection and subsequently used for RNA-seq and RNA BS-seq library construction.

### RNA BS-seq library construction

RNA bisulfite conversion was performed as previously described [68] with minor modifications. Briefly, poly(A) RNA was first mixed with spiked-in Xef1 unmethylated RNA at a ratio of 0.5%. The spiked-in unmethylated mRNA was transcribed from the pTRI-Xef plasmid supplied by the MEGAscript™ T7 Transcription Kit (Invitrogen, cat# AM1333), which encodes the 1.85-kb Xenopus elongation factor 1α mRNA according to the manufacturer’s manual. Briefly, the linearized pTRI-Xef plasmid was transcribed in vitro in a reaction with MEGAscript T7 RNA polymerase (Ambion) at 37 °C for 4 h, followed by DNase treatment at 37 °C for 15min to remove the DNA template and then purified using the RNeasy Mini Kit (QIAGEN, cat# 74104). RNA bisulfite conversion was performed with an initial denaturation at 95°C for 1min, followed by three cycles of 70°C for 10min and 64°C for 45min using the EZ RNA methylation Kit (Zymo Research, cat# R5001). The bisulfite-converted RNA was subjected to the stranded RNA-seq library construction procedure using the TruSeq Stranded mRNA Library Preparation Kit (Illumina, cat# RS-122-2101). We modified the procedure to skip the RNA fragmentation step and to supply both random and ACT random hexamers during the first strand cDNA synthesis.

### RNA BS-seq data analysis

The RNA BS-seq data analysis was performed as previously described [64]. The adapter sequences, 6bp from the 5′ and 3′ ends of both the forward and reverse reads, low-quality bases, and polyXs with a threshold of a 10-base nucleotide repeat were removed from raw reads using fastp v0.20 [69]. The processed reads with lengths greater than 50bp were defined as clean reads and mapped to the mouse genome mm10 using meRanGh of the meRanTk package [70]. Bisulfite conversion rates were estimated according to the Xef spike-in controls. Uniquely aligned reads were used to call candidate m^5^Cs by meRanCall package from meRanTK software. To achieve high confidence in methylation calling, a sequential filtering pipeline was applied to each site. First, a 3C filter was performed, that is, bisulfite-converted reads with more than 3 unconverted Cs were considered as incomplete conversions and, thus, were filtered (3C filter). Then, a standard filter was applied: (1) coverage depth ≥ 20, (2) methylation level ≥ 0.1, and (3) methylated cytosine depth ≥ 6. After the standard filter, the m^5^C sites with “signal/noise” ratios greater than 0.9 [43, 49] and FDR-adjusted *p*-values less than 0.05 [68, 70] were retained. Lastly, RNAfold of the ViennaRNA v2.2.9 software (–maxBPspan 150, -T 70, –MEA 0.1) was applied to predict conversion-resistant regions [71]. m^5^C sites located in these regions were further removed. After all the filtering steps, the leftover m^5^C sites that were present in both biological replicates were considered high-confidence m^5^C sites.

The m^5^C sites were annotated using a custom script and the Ensembl mm10 v79 GTF and assigned to 5′ UTR, 3′ UTR, CDS, and noncoding RNA. According to the average lengths of 5′ UTR, 3′ UTR, and CDS in the whole transcriptome, the three segments were divided into 5, 18, and 22 bins, respectively. The numbers of the m^5^C sites in each bin were counted and the percentage was calculated to plot the distribution of m^5^C sites along the mRNA transcripts.

### Differential methylation analysis

Differential methylation analysis was performed on m^5^C sites that met the following two criteria: (1) coverage depth ≥ 20 in the libraries involved in the comparison, and (2) they were methylated in at least one condition. Fisher’s exact test was used to evaluate the significance of differential methylation and the FDR method was applied to correct multiple comparisons. Sites with adjusted *p*-values < 0.05 were considered as differentially methylated sites (DMS).

### RNA-seq library construction

Stranded RNA-seq libraries were constructed using the TruSeq Stranded mRNA Library Preparation Kit (Illumina, cat# RS-122-2101) following the manufacturer’s instructions. Briefly, after two rounds of poly(A) selection, the mRNA samples were fragmented and primed to synthesize first strand cDNA, followed by synthesis of the second strand cDNA. After Ampure XP bead purification, dA tailing was performed and indexed adapters were ligated to both ends of the ds cDNA. Adapter-ligated DNA fragments were enriched by PCR amplification for 12 cycles. After Ampure XP bead purification, the PCR products were size-selected with a range from 350 to 550bp on 2% dye-free agarose gel using the pippin recovery system (Sage Science). The recovered libraries were sequenced on a Hiseq 4000 platform in the 150-bp paired end mode (Illumina).

### RNA-seq data analysis

Adapter sequences and low-quality bases (*Q* < 30) were removed from raw reads using Trim Galore (version 0.5.0) (https://www.bioinformatics.babraham.ac.uk/projects/trim_galore/). Processed reads with lengths greater than 30 nt were defined as clean reads. Clean reads were mapped to the mm10 genome and gene expression levels were outputted by RSEM [72]. The union set of the two replicates were compiled as the list of genes expressed. The raw counts were employed to identify differential expression genes by DESeq2 [48]. Differentially expressed genes were defined as ones with a fold change greater than 1.2 and an adjusted *p*-value less than 0.05.

### Differential translation analysis

Translation efficiency (TE) was estimated as the ratio between polysome mRNA counts and total mRNA counts (TE=polysome/total). Fold changes in TE between two conditions were calculated as TE (treatment)/TE (control). Differential translation efficiency analysis was performed using the Xtail package [53] with the following parameter: minMeanCount = 1. Differentially translated genes (DTGs) were defined as ones with a fold change greater than 1.2 and an adjusted *p*-value less than 0.05.

### Gene Ontology (GO) analysis

GO analysis was performed using DAVID (Database for Annotation, Visualization and Integrated Discovery) [73]. Default parameters were used for the enrichment analysis for Biological Process (BP), cellular component (CC), and molecular function (MF). The representative GO terms were shown.

### Immunostaining

Immunostaining was performed as previously described [74]. Briefly, mouse neural stem cells were seeded on an 8-well chamber overnight. The NSCs were fixed with 4% paraformaldehyde in PBS at room temperature (RT) for 15 min. After washing three times with PBS, NSCs were permeabilized with 0.2% Triton X-100 in PBS at RT for 10 min. The cells were then blocked with 5% normal goat serum (Thermo Fisher, cat# 50062Z) at RT for 1 h and incubated with mouse anti-Nestin antibody (Millipore, cat# MAB353) and rabbit anti-Sox2 antibody (Abcam, cat# ab97959) at 4 °C overnight. After washing three times with 1×PBS, the cells were incubated with Cy3-conjugated anti-rabbit IgG (Invitrogen, cat# A10520) and Alexa Fluor 488-conjugated anti-mouse IgG (Invitrogen, cat# A10680) secondary antibodies at RT in darkness for 1 h. After washing three times with 1×PBS, cells were mounted with DAPI-Fluoromount-G™ Clear Mounting Media (SouthernBiotech, cat# 010020) and the fluorescent images were captured using a confocal microscope.

### RT-qPCR

RT-qPCR was performed as previously described [74]. Briefly, 1 μg total RNA was reverse transcribed to cDNA using the high-capacity cDNA reverse transcription kit (Applied Biosystems, cat# 43-688-14). qPCR reaction was performed using GoTaq qPCR Master Mix (Promega, cat# A6001) on Step One Plus Real-Time PCR Systems. mRNA expression levels were determined using the ∆∆Ct method with GAPDH as an internal reference control.

### Western blot

NSCs were lysed by RIPA buffer supplemented with a protease inhibitor cocktail (Thermo Scientific). The total protein samples were loaded to run SDS-PAGE on the NuPAGE 4 to 12%, Bis-Tris mini protein gel (Thermo Fisher, cat# NP0335BOX), and transferred to the nitrocellulose membrane (Thermo Fisher, cat# LC2006). After blocking in 5% milk-PBST, the membrane was incubated with primary antibodies at 4 °C overnight, followed by incubation with secondary antibodies at room temperature for 1h, with washing steps in between. The membrane was developed with Super Signal West Pico PLUS Chemiluminescent Substrate (Thermo Fisher, cat# 34580), and the images were captured using the Bio Rad ChemiDoc imaging system. The primary antibodies used were rabbit anti-Ndufa13 antibody (Proteintech, cat# 10986-1-AP), rabbit anti-Gng11 antibody (Thermo Fisher, cat# PA5-100666), mouse anti-Ptx3 antibody (Santa Cruz, cat# sc-373951), and rabbit anti-GAPDH antibody (CST, cat# 2118).

## Supplementary Information

**Additional file 1: Figure S1.** Quality control of total mRNA-seq datasets. (a, b, c) Scatter plot showing the Pearson correlation between two biological replicates in total mRNA-seq datasets in NSCs. (d) Principal component analysis of total mRNA-seq datasets. **Figure S2.** Validation of differentially expressed genes (DEGs). (a-g) RT-qPCR was performed to validate the expression of DEGs identified by RNA-seq. (*<0.05, **<0.01, ***<0.001.) Numerical data is provided in Additional file 7. The error bars indicate standard error of the mean (SEM). **Figure S3.** Distribution of m^5^C sites in total mRNA BS-seq datasets. (a) Pie chart showing the categories of RNA molecules with m^5^C modification. (b) Histogram showing the distribution of methylation level for each condition. **Figure S4.** Quality control of polysome mRNA-seq datasets. (a, b, c) Scatter plot showing the Pearson correlation between two biological replicates in polysome mRNA-seq datasets in NSCs. (d) Principal component analysis of polysome mRNA-seq datasets. **Figure S5.** Validation of differentially translated genes (DTGs). (a, b) Western blot was performed to validate the expression of DTGs identified by polysome profiling. Gng11 showed increased translation efficiency in LF (a), and Ptx3 showed increased translation efficiency in HF (b). Original blot images were provided in Additional file 8. **Figure S6.** Characterization of m^5^C sites in polysome mRNA BS-seq datasets. (a) Venn diagram showing the overlap of m^5^C sites between two replicates. (b) Dot plot showing the methylation level correlation of the overlapped m^5^C sites between two replicates. (c) Box plot showing the methylation level of overlapped and non-overlapped m^5^C sites in the two replicates. (d) Pie chart showing the categories of RNA molecules with m^5^C modification. (e) Histogram showing the distribution of methylation level for each condition. **Figure S7.** Functional annotation of m^5^C-modified mRNAs in polysome mRNAs. (a) Venn diagram showing the overlap of m^5^C sites among the three conditions (pLF, pMF, pHF) (b) Bubble plot showing the GO terms of m^5^C-modified mRNAs in pLF, pMF and pHF. **Figure S8.** Comparison between total and polysome methylomes. (a) Venn diagram showing the overlap of m^5^C sites between total and polysome mRNAs. (b) Box plot showing the methylation level of the union of m^5^C sites between total and polysome mRNAs with 20x coverage in both samples. A Wilcoxon signed-rank test was performed. **Figure S9.** Visualization of the correlation between m5C methylation and mRNA translation. (a, b, c) Left panel depicts transcript-level read pileup of the mRNAs: Hdgf (a), Glg1 (b), and Dgcr2 (c) using the IGV browser. The total and polysome mRNA-seq datasets were shown (green – LF, blue – MF, red –HF). Right panel depicted the methylation level of the m5C site identified in the respective transcript in both total and polysome mRNAs (t: total, p: polysome).

**Additional file 2: Table 1.** Library statistics.

**Additional file 3: Table 2.** RNA expression level of total and polysome mRNA-seq libraries.

**Additional file 4: Table 3.** List of differentially expressed genes.

**Additional file 5: Table 4.** List of differentially translated genes.

**Additional file 6: Table 5.** List of high-confidence m^5^C sites in mouse NSCs.

**Additional file 7.** Combined raw data, with each figure on a spreadsheet.

**Additional file 8.** Original uncropped blot images.

**Additional file 9.** Source codes used to generate figures.

## Data Availability

All data generated or analyzed during this study are included in this published article, its supplementary information files, and publicly available repositories. Sequencing data generated in this study have been deposited to the NCBI Gene Expression Omnibus under accession number GSE151726. Analyses in this study were performed using the R v4.1.1, and Python 3.9.4 packages Biopython v1.78, matplotlib v3.3.4, Seaborn v0.11, and Pysam v0.16. Individual data values are provided in Additional file 7. Source codes are provided in Additional file 9. Source codes and input data used to generate figures were provided via GitHub link: https://github.com/zaustinj33/RNABSseq_FolicAcid

## Abbreviations

NSC: Neural stem cell
NTD: Neural tube defect
AD: Alzheimer’s disease
ASD: Autism spectrum disorder
P1: Postnatal day 1
tRNA: Transfer RNA
rRNA: Ribosomal RNA
mRNA: Messenger RNA
mt-mRNA: Mitochondrial mRNA
LF: Low folate concentration
MF: Median folate concentration
HF: High folate concentration
TPM: Transcripts per million
DEG: Differentially expressed gene
DMS: Differentially methylated m5C site
GO annotation: Gene Ontology annotation
DTG: Differentially translated gene
