# Supplementary material for: Folate regulates RNA m5C modification and translation in neural stem cells
Source: BMC Biol. 2022 Nov 23;20:261. doi: 10.1186/s12915-022-01467-0 (PMC9686110; doi:10.1186/s12915-022-01467-0)
Supplement: Supplementary file 1 — Additional file 1: Figure S1. Quality control of total mRNA-seq datasets. (a, b, c) Scatter plot showing the Pearson correlation between two biological replicates in total mRNA-seq datasets in NSCs. (d) Principal component analysis of total mRNA-seq datasets. Figure S2. Validation of differentially expressed genes (DEGs). (a-g) RT-qPCR was performed to validate the expression of DEGs identified by RNA-seq. (*<0.05, **<0.01, ***<0.001.) Numerical data is provided in Additional file 7. The error bars indicate standard error of the mean (SEM). Figure S3. Distribution of m5C sites in total mRNA BS-seq datasets. (a) Pie chart showing the categories of RNA molecules with m5C modification. (b) Histogram showing the distribution of methylation level for each condition. Figure S4. Quality control of polysome mRNA-seq datasets. (a, b, c) Scatter plot showing the Pearson correlation between two biological replicates in polysome mRNA-seq datasets in NSCs. (d) Principal component analysis of polysome mRNA-seq datasets. Figure S5. Validation of differentially translated genes (DTGs). (a, b) Western blot was performed to validate the expression of DTGs identified by polysome profiling. Gng11 showed increased translation efficiency in LF (a), and Ptx3 showed increased translation efficiency in HF (b). Original blot images were provided in Additional file 8. Figure S6. Characterization of m5C sites in polysome mRNA BS-seq datasets. (a) Venn diagram showing the overlap of m5C sites between two replicates. (b) Dot plot showing the methylation level correlation of the overlapped m5C sites between two replicates. (c) Box plot showing the methylation level of overlapped and non-overlapped m5C sites in the two replicates. (d) Pie chart showing the categories of RNA molecules with m5C modification. (e) Histogram showing the distribution of methylation level for each condition. Figure S7. Functional annotation of m5C-modified mRNAs in polysome mRNAs. (a) Venn diagram showing the overlap of [file 12915_2022_1467_MOESM1_ESM.pdf]

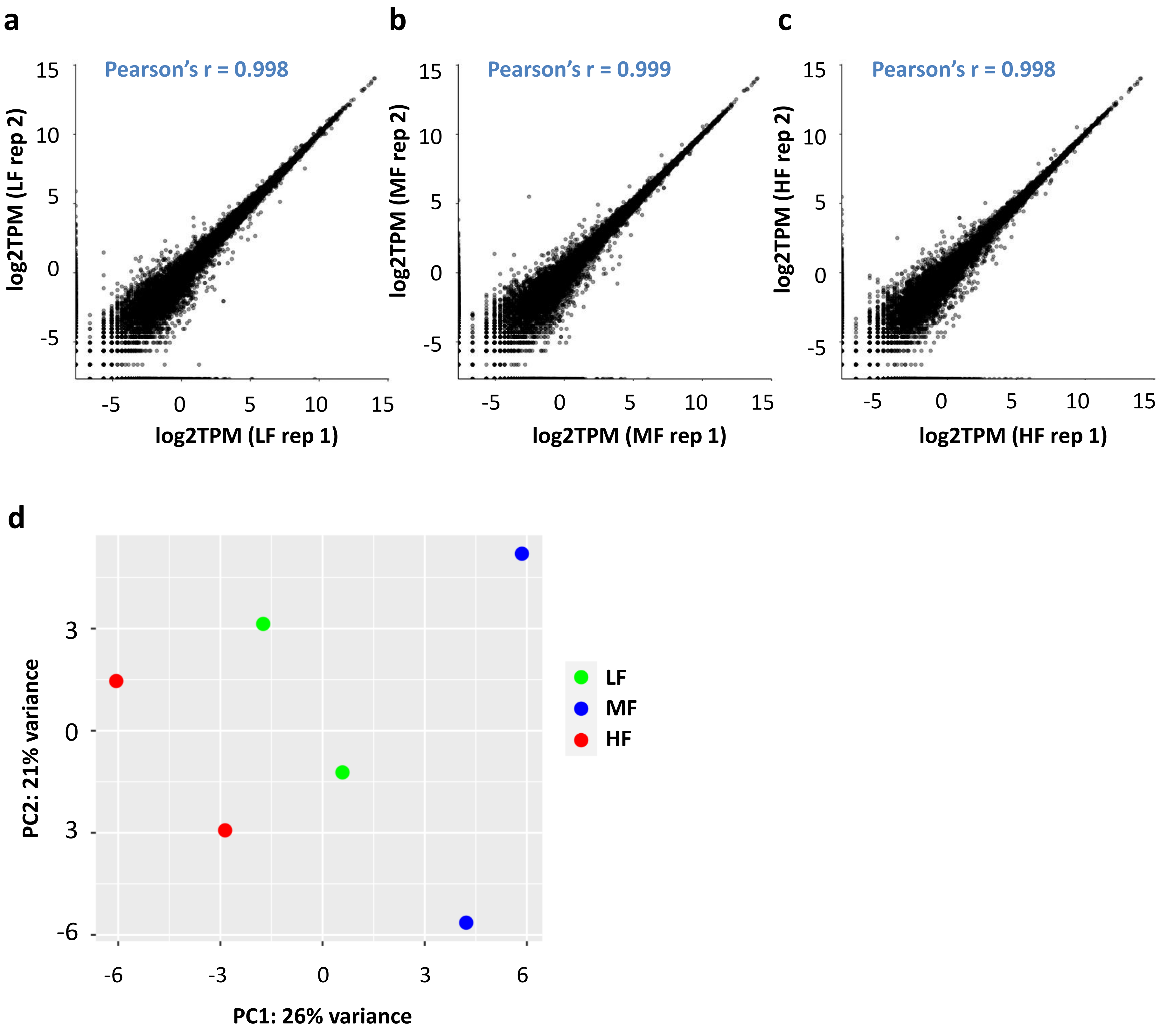

Fig. S1. Quality control of total mRNA-seq datasets. (a, b, c) Scatter plot showing the Pearson correlation between two biological replicates in total mRNA-seq datasets in NSCs. (d) Principal component analysis of total mRNA-seq datasets.

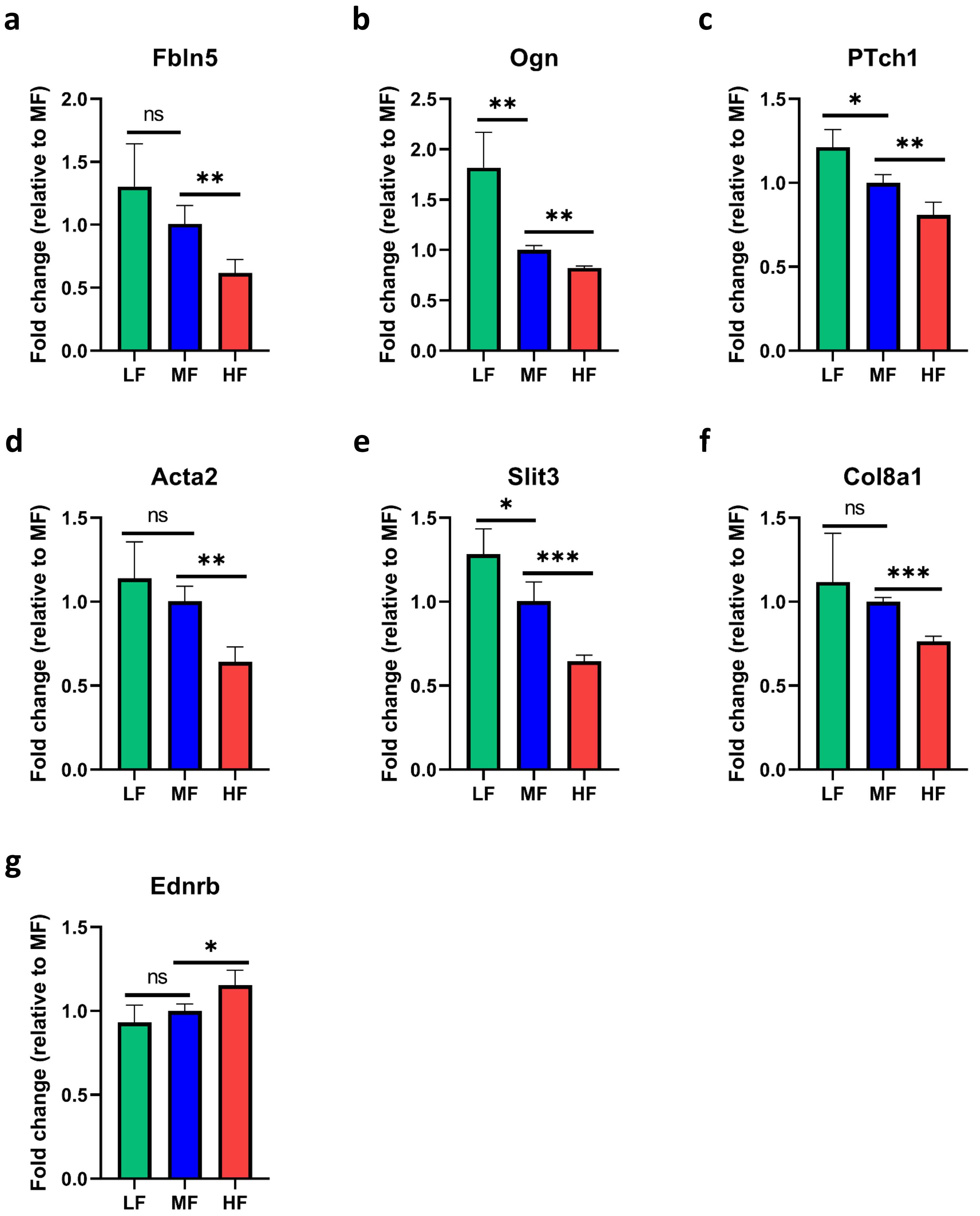

Fig. S2. Validation of differentially expressed genes (DEGs). (a-g) RT-qPCR was performed to validate the expression of DEGs identified by RNA-seq. (number of replicates = 3 or 4. *<0.05, **<0.01, ***<0.001.) Numerical data is provided in Additional file 7. The error bars indicate standard error of the mean (SEM).

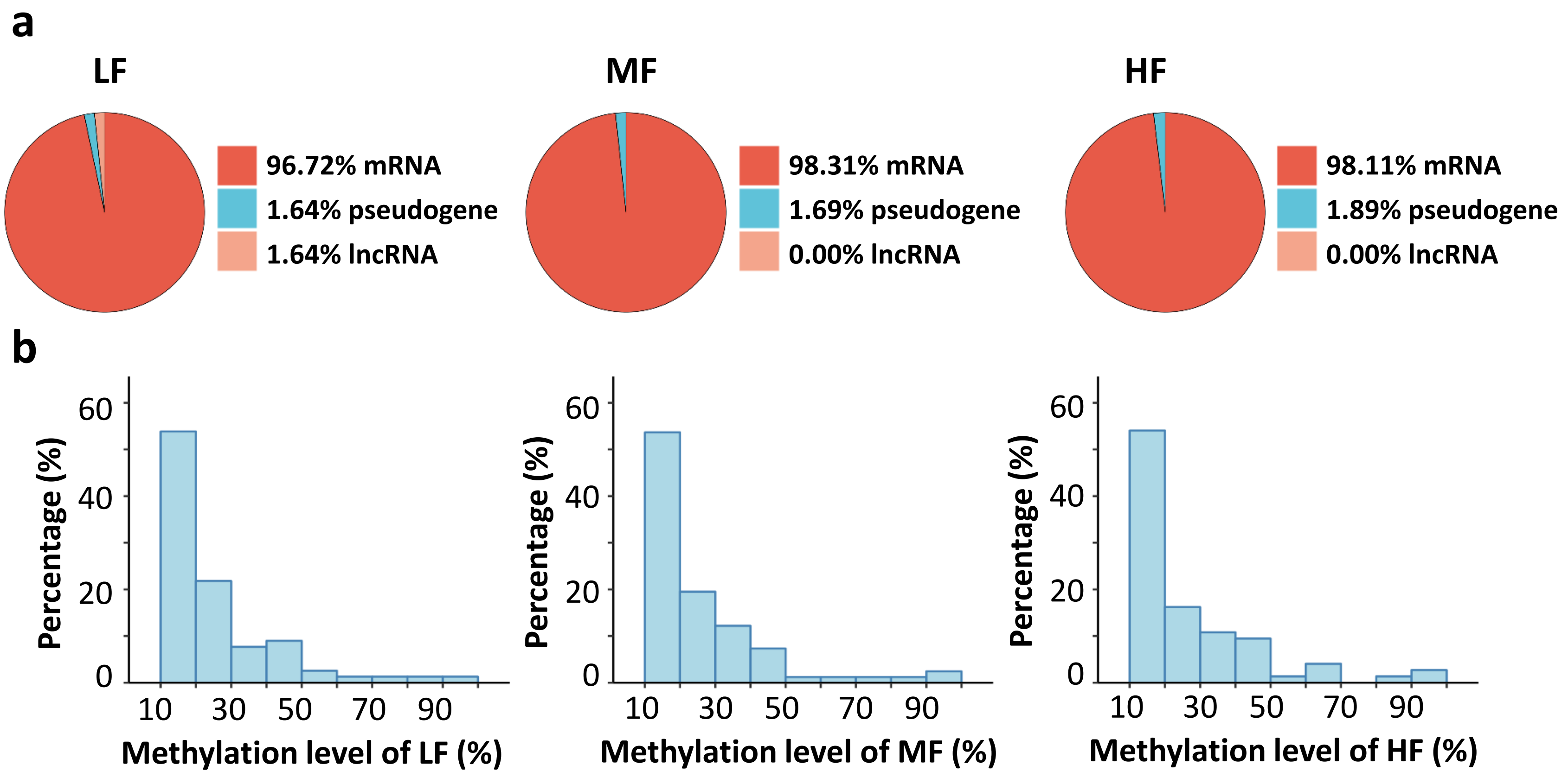

Fig. S3. Distribution of m^{5}C sites in total mRNA BS-seq datasets. (a) Pie chart showing the categories of RNA molecules with m^{5}C modification. (b) Histogram showing the distribution of methylation level for each condition.

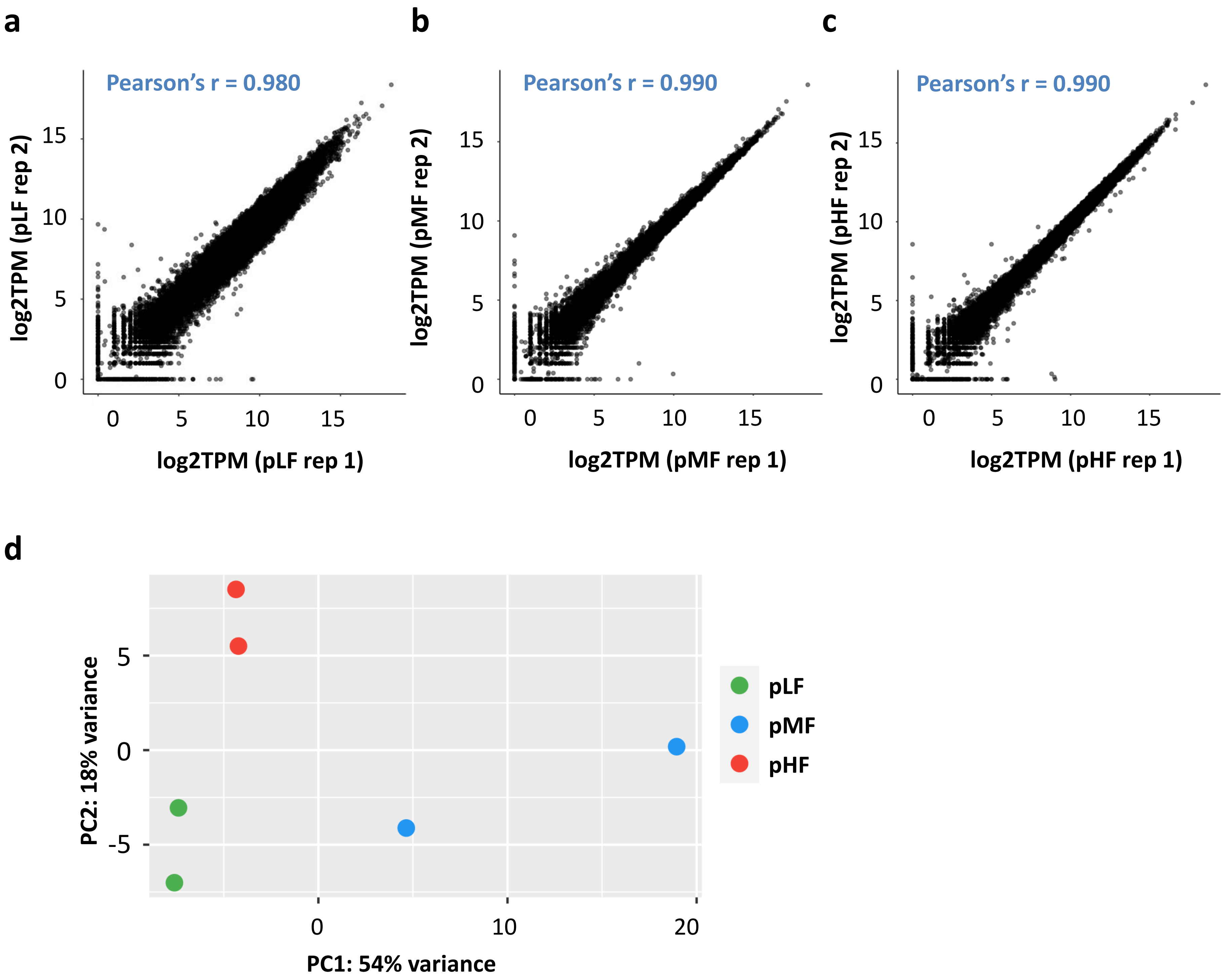

Fig. S4. Quality control of polysome mRNA-seq datasets. (a, b, c) Scatter plot showing the Pearson correlation between two biological replicates in polysome mRNA-seq datasets in NSCs. (d) Principal component analysis of polysome mRNA-seq datasets.

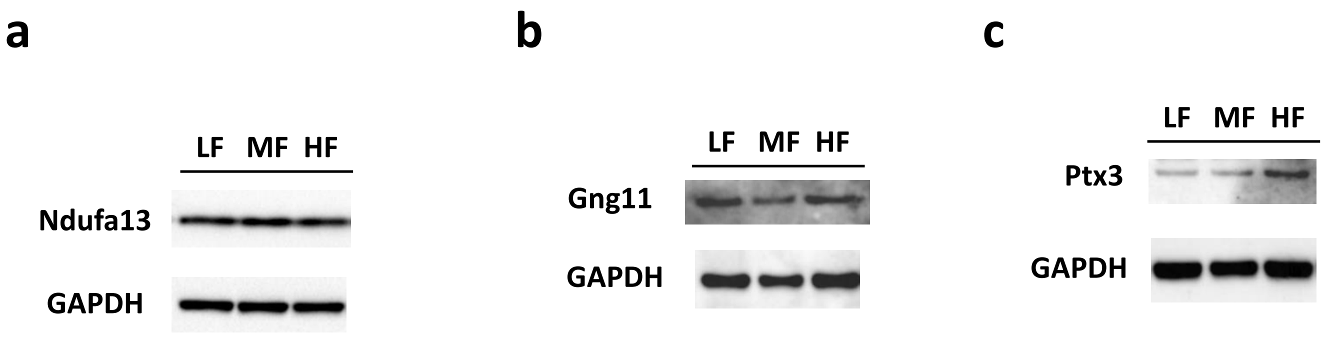

Fig. S5. Validation of differentially translated genes (DTGs). (a, b) Western blot was performed to validate the expression of DTGs identified by polysome profiling. Ndufa13 showed decreased translation efficiency in LF (a), Gng11 showed increased translation efficiency in LF (b), and Ptx3 showed increased translation efficiency in HF (c). Original blot images were provided in Additional file 8.

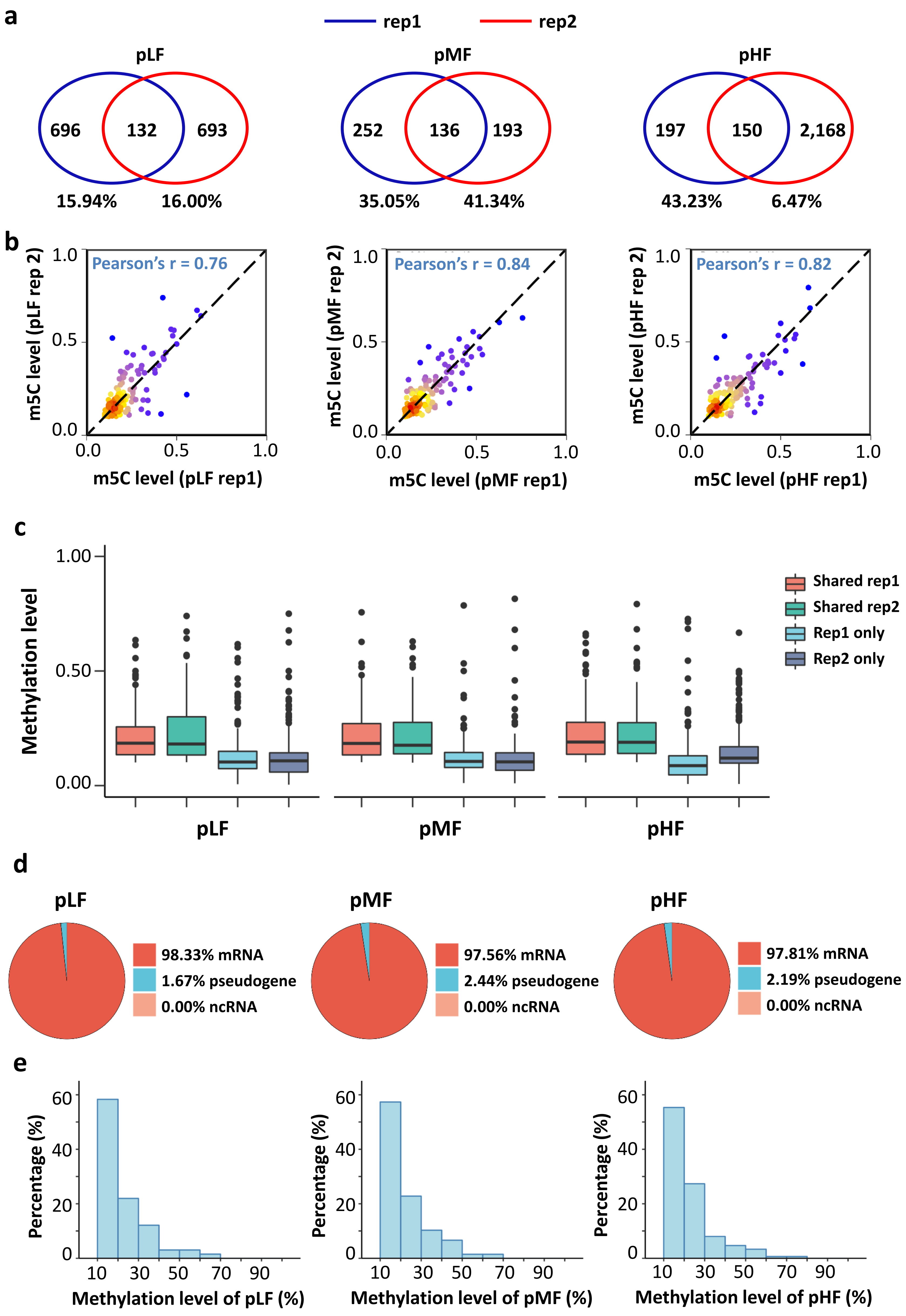

a
rep1
rep2
pLF
696
132
693
15.94%
16.00%
pMF
252
136
193
35.05%
41.34%
pHF
197
150
2,168
43.23%
6.47%
b
Pearson's r = 0.76
m5C level (pLF rep 2)
m5C level (pLF rep1)
Pearson's r = 0.84
m5C level (pMF rep 2)
m5C level (pMF rep1)
Pearson's r = 0.82
m5C level (pHF rep 2)
m5C level (pHF rep1)
c
Methylation level
Shared rep1
Shared rep2
Rep1 only
Rep2 only
pLF
pMF
pHF
d
pLF
98.33% mRNA
1.67% pseudogene
0.00% ncRNA
pMF
97.56% mRNA
2.44% pseudogene
0.00% ncRNA
pHF
97.81% mRNA
2.19% pseudogene
0.00% ncRNA
e
Percentage (%)
Methylation level of pLF (%)
Methylation level of pMF (%)
Methylation level of pHF (%)

Fig. S6. Characterization of m^5C sites in polysome mRNA BS-seq datasets. (a) Venn diagram showing the overlap of m^5C sites between two replicates. (b) Dot plot showing the methylation level correlation of the overlapped m^5C sites between two replicates. (c) Box plot showing the methylation level of overlapped and non-overlapped m^5C sites in the two replicates. (d) Pie chart showing the categories of RNA molecules with m^5C modification. (e) Histogram showing the distribution of methylation level for each condition.

a

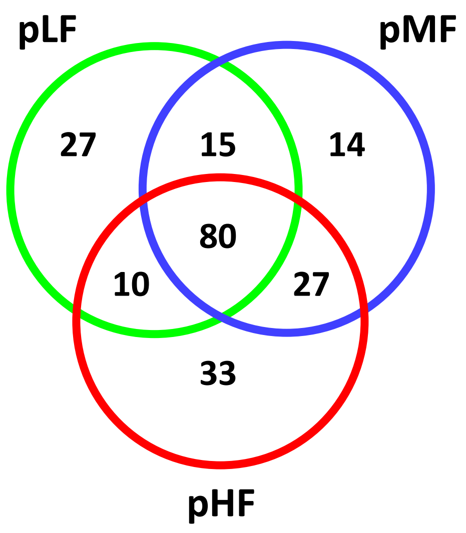

b

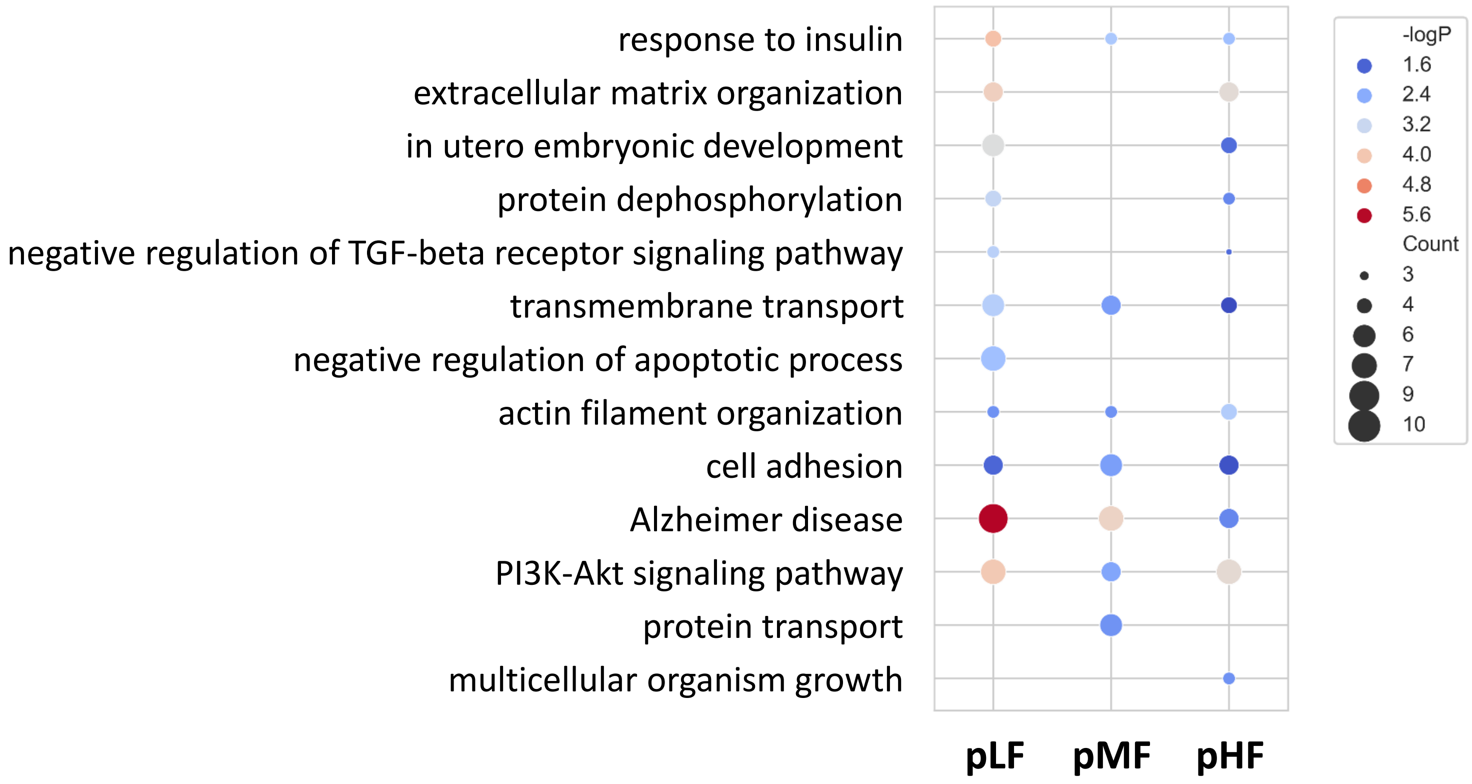

Fig. S7. Functional annotation of m^{5}C-modified mRNAs in polysome mRNAs. (a) Venn diagram showing the overlap of m^{5}C sites among the three conditions (pLF, pMF, pHF) (b) Bubble plot showing the GO terms of m^{5}C-modified mRNAs in pLF, pMF and pHF.

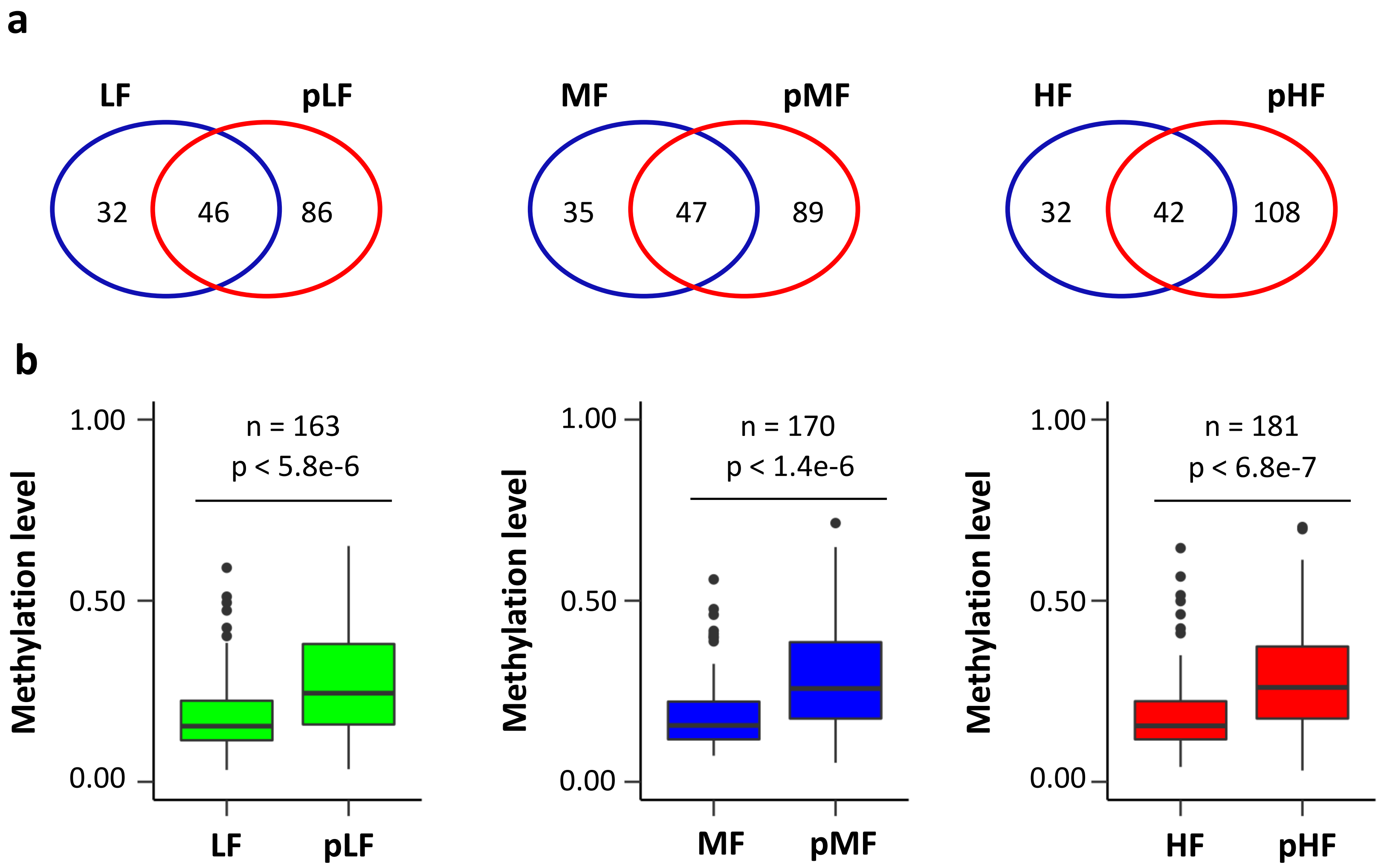

Fig. S8. Comparison between total and polysome methylomes. (a) Venn diagram showing the overlap of m^5C sites between total and polysome mRNAs. (b) Box plot showing the methylation level of the union of m^5C sites between total and polysome mRNAs with 20x coverage in both samples. A Wilcoxon signed-rank test was performed.

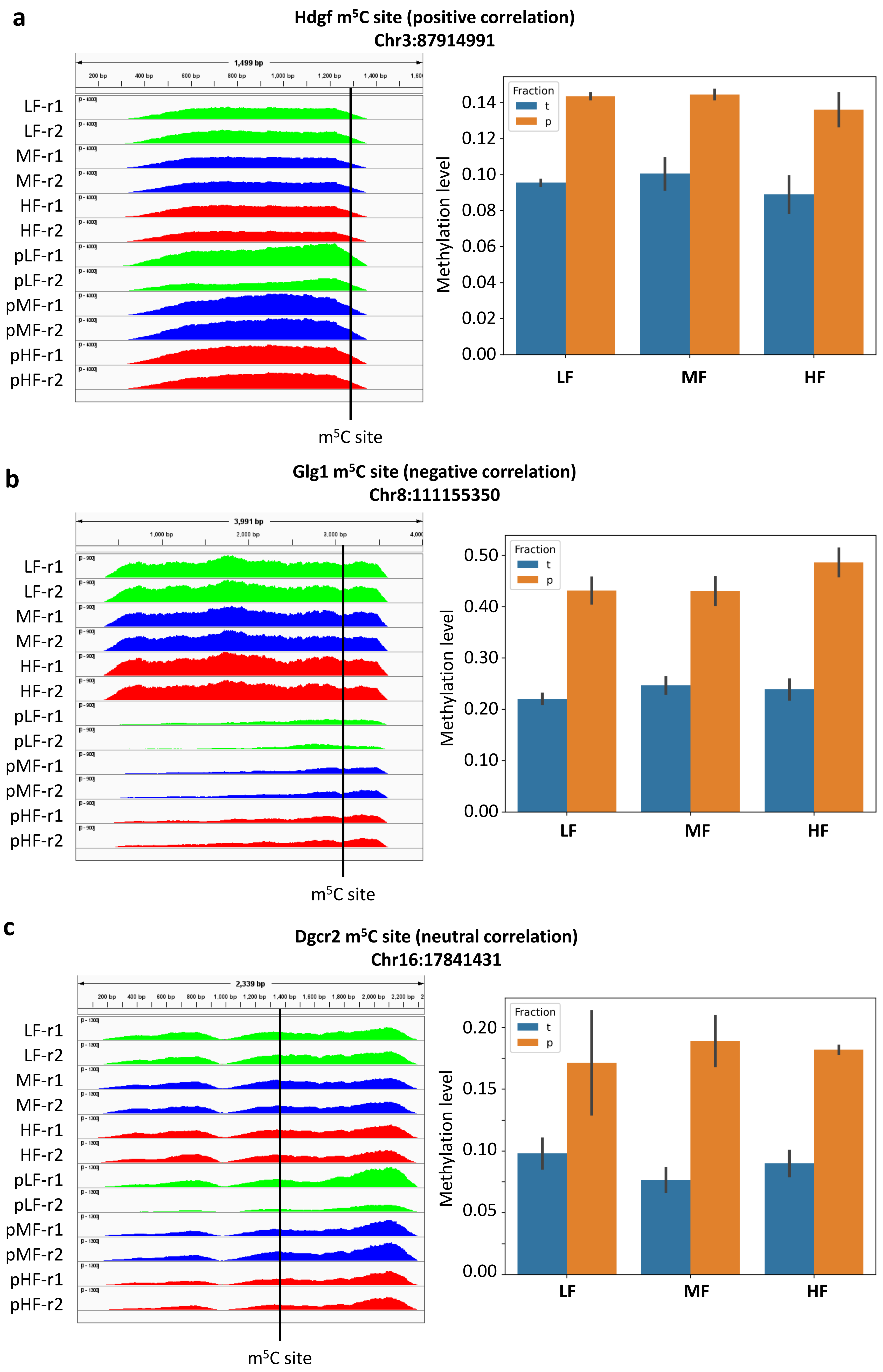
a
Hdgf m5C site (positive correlation)
Chr3:87914991
1,499 bp
LF-r1
LF-r2
MF-r1
MF-r2
HF-r1
HF-r2
pLF-r1
pLF-r2
pMF-r1
pMF-r2
pHF-r1
pHF-r2
m5C site
Fraction
t
p
Methylation level
0.00
0.02
0.04
0.06
0.08
0.10
0.12
0.14
LF
MF
HF
b
Glg1 m5C site (negative correlation)
Chr8:111155350
3,991 bp
LF-r1
LF-r2
MF-r1
MF-r2
HF-r1
HF-r2
pLF-r1
pLF-r2
pMF-r1
pMF-r2
pHF-r1
pHF-r2
m5C site
Fraction
t
p
Methylation level
0.00
0.10
0.20
0.30
0.40
0.50
LF
MF
HF
c
Dgcr2 m5C site (neutral correlation)
Chr16:17841431
2,339 bp
LF-r1
LF-r2
MF-r1
MF-r2
HF-r1
HF-r2
pLF-r1
pLF-r2
pMF-r1
pMF-r2
pHF-r1
pHF-r2
m5C site
Fraction
t
p
Methylation level
0.00
0.05
0.10
0.15
0.20
LF
MF
HF

Fig. S9. Visualization of the correlation between m^5C methylation and mRNA translation. (a, b, c) Left panel depicts transcript-level read pileup of the mRNAs: Hdgf (a), Glg1 (b), and Dgcr2 (c) using the IGV browser. The total and polsyome mRNA-seq datasets were shown (green – LF, blue – MF, red –HF). Right panel depicted the methylation level of the m^5C site identified in the respective transcript in both total and polysome mRNAs (t: total, p: polysome).
