# Supplementary figures and images for: Folate regulates RNA m5C modification and translation in neural stem cells
Source: BMC Biol. 2022 Nov 23;20:261. doi: 10.1186/s12915-022-01467-0 (PMC9686110; doi:10.1186/s12915-022-01467-0)

Additional file 8: original uncropped blot images (related to Fig. S5).

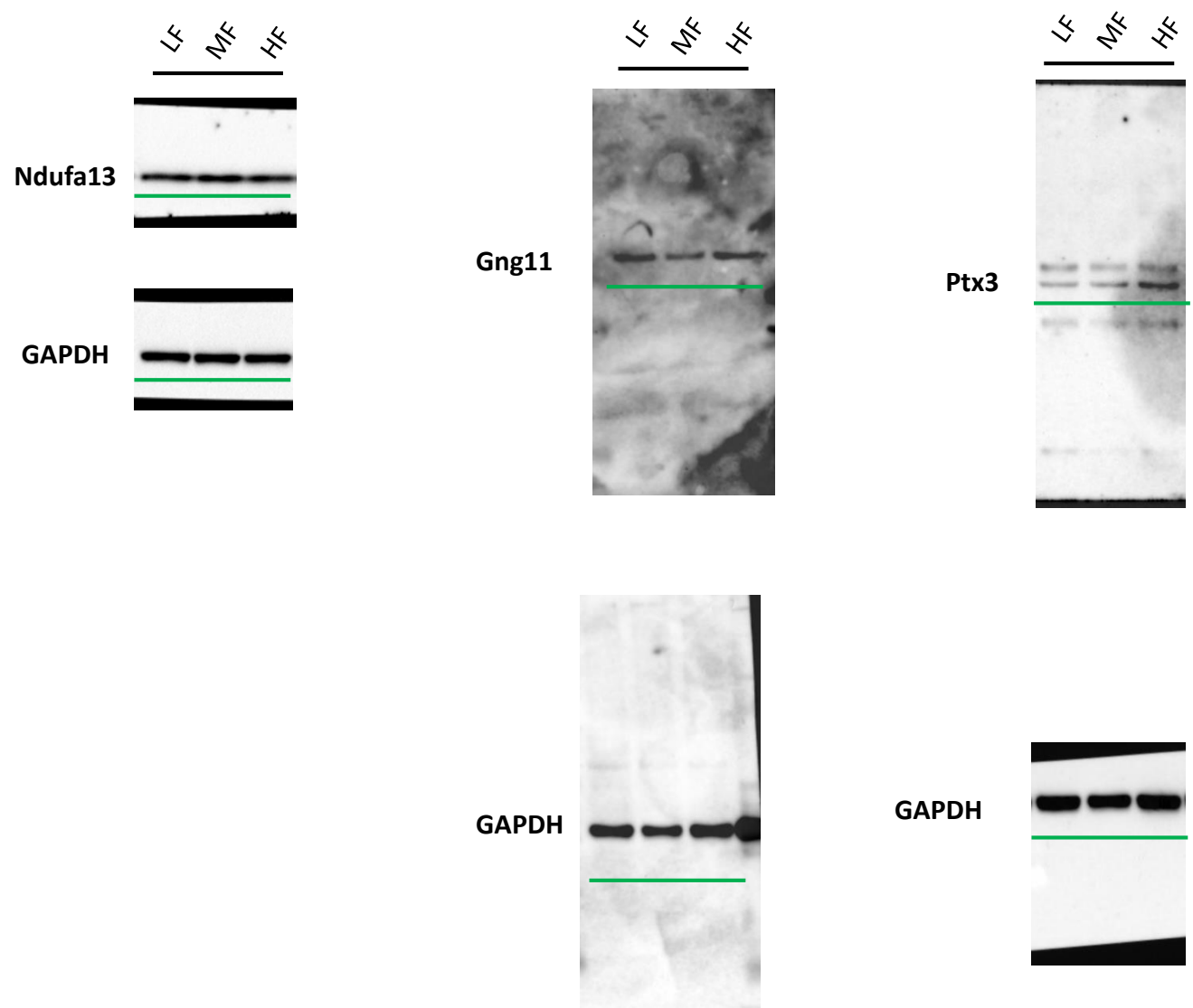

Supplement: Supplementary file 8 — Additional file 8. Original uncropped blot images. [file 12915_2022_1467_MOESM8_ESM.pdf]
